# Evaluating Comorbidity Scores in Geriatric Ovarian Cancer: A Retrospective Cohort Analysis

**DOI:** 10.3390/medicina62010189

**Published:** 2026-01-16

**Authors:** Simay Cokgezer, Naziye Ak, Muhammet Senkal, Aysel Safaraliyeva, Didem Tastekin, Pınar Mualla Saip

**Affiliations:** 1 Department of Medical Oncology, Institute of Oncology, Istanbul University, Millet Street, Çapa Campus, Fatih, Istanbul 34093, Türkiye; aknaziyeak@gmail.com (N.A.); didem_doktor@hotmail.com (D.T.); pinarsaip@gmail.com (P.M.S.); 2 Department of Internal Medicine, Istanbul Faculty of Medicine, Istanbul University, Millet Street, Çapa Campus, Fatih, Istanbul 34093, Türkiye; mamulli.muhammetsenkal@gmail.com (M.S.); ayselsafaraliyeva19@gmail.com (A.S.)

**Keywords:** Charlson Comorbidity Index (CCI), comorbidity assessment, elderly ovarian cancer, geriatric oncology

## Abstract

*Background and Objectives:* This study aimed to comparatively evaluate the association of commonly used comorbidity scores with survival, mortality, and recurrence in ovarian cancer patients aged 50 years and above. *Materials and Methods:* In this single-center, retrospective study, 130 female patients diagnosed between 2017 and 2024 who had received systemic therapy and had complete medical records were included. Comorbidity scores—including the Charlson Comorbidity Index (CCI), Cumulative Illness Rating Scale-Geriatric (CIRS-G), Adult Comorbidity Evaluation-27 (ACE-27), Elixhauser Comorbidity Index, Index of Coexistent Disease (ICED), and Functional Comorbidity Index (FCI)—were calculated for each patient. Survival analyses were conducted using the Kaplan–Meier method and Cox regression modeling. The prognostic accuracy of comorbidity scores was assessed via receiver operating characteristic (ROC) curve analysis. *Results:* Patients with higher CCI scores had significantly shorter survival, and CCI was identified as an independent prognostic factor in multivariate analysis. While other comorbidity scores were associated with overall survival in univariate analyses, they lost statistical significance in multivariate models. Patients with a higher comorbidity burden experienced more frequent disease recurrence and shorter time to recurrence. *Conclusions:* Comorbidity burden is a key clinical determinant of survival and disease trajectory in older patients with ovarian cancer. The CCI demonstrated the highest prognostic accuracy in this population and may serve as a valuable tool in individualized treatment planning. Integration of comorbidity-based assessments into standard decision-making processes is recommended in geriatric oncology practice.

## 1. Introduction

According to GLOBOCAN 2022 data, the incidence and mortality rates of ovarian cancer increase markedly with age. Among women aged 50 and above, the incidence is reported as 27.6 per 100,000 and the mortality rate as 17.6 per 100,000. In contrast, among women under 50 years of age, the incidence and mortality rates are 6.0 and 2.7 per 100,000, respectively. These figures highlight the significantly elevated risk of ovarian cancer and cancer-related death in the older population [1].

Standard treatment for ovarian cancer typically consists of primary cytoreductive surgery followed by platinum-based chemotherapy. However, in advanced-stage disease where optimal cytoreduction is unlikely or in patients for whom surgery poses excessive risk due to comorbidities, interval cytoreductive surgery following neoadjuvant chemotherapy has been shown to be beneficial [2].

Treating elderly patients presents significant challenges. The often-complex medical histories common in this age group can limit the feasibility of aggressive surgical interventions. In addition, concomitant medication use and age-related physiological changes contribute to increased rates of chemotherapy-related toxicity, which may lead to treatment delays, dose reductions, or, in some cases, the inability to administer standard therapeutic regimens [3].

When planning treatment for older patients, the goal should not be limited to prolonging overall survival (OS) and progression-free survival (PFS), but should also include consideration of physical status, social support systems, and overall quality of life. In frail patients, personalized treatment strategies with lower toxicity, reduced invasiveness, and flexible dosing schedules are recommended [4]. To improve treatment outcomes in older women diagnosed with ovarian cancer, a better understanding of age-related differences in tumor biology and the development of decision-support tools that can accurately identify patients capable of tolerating cytoreductive surgery and chemotherapy are essential [5].

Comprehensive pre-treatment assessment in elderly ovarian cancer patients is critical for determining appropriate therapeutic strategies. However, there is currently no consensus in the literature regarding the criteria that should guide this evaluation process. In this study, we aimed to investigate the clinical and geriatric factors that may influence treatment response, toxicity, and survival. Specifically, we sought to compare the predictive value of commonly used comorbidity indices—including the ECOG PS, CCI, CIRS-G, ACE-27, Elixhauser Comorbidity Index, ICED, and FCI—in relation to treatment-related toxicity and survival outcomes.

## 2. Materials and Methods

This study was designed as a retrospective, observational, single-center cohort analysis. Ethical approval was obtained from the Clinical Research Ethics Committee of Istanbul Faculty of Medicine on 5 March 2025. The medical records of female patients aged 50 years and above, who were diagnosed with ovarian cancer and received follow-up and treatment at our institution between 1 January 2017, and 1 December 2024, were retrospectively reviewed.

Inclusion criteria were being aged 50 or older at the time of diagnosis, having received systemic therapy (chemotherapy and/or targeted therapy), and having complete medical records available. Exclusion criteria included patients who met the age and diagnosis criteria but whose medical records were inaccessible. All data were anonymized and collected through review of patient files and the institutional digital archive system. Data collected included demographic information (age, height, weight, body mass index, menopausal status), clinical characteristics (ECOG performance status, smoking and alcohol use, presence of comorbidities), treatment-related details (surgical procedures, chemotherapy regimens, use of targeted agents, and radiotherapy), and follow-up data (recurrence, duration of follow-up, vital status). All information was recorded using standardized data collection forms.

Adverse events related to treatment were classified according to the Common Terminology Criteria for Adverse Events (CTCAE) version 5.0, while treatment responses were assessed based on RECIST 1.1 criteria. Disease staging was performed according to the FIGO 2017 (8th edition) classification. Progression-free and overall survival durations were calculated from the date of diagnosis to the date of progression or death, whichever occurred first.

To evaluate comorbidity burden, seven different comorbidity indices were used in the study: the CCI, CIRS-G, ACE-27, Elixhauser, ICED, FCI and Elixhauser. Each score was calculated individually for every patient and analyzed by stratifying patients according to appropriate cutoff values.

The primary endpoint of the study was to evaluate the relationship between comorbidity scores and mortality in ovarian cancer patients aged 50 and above. In this context, death was considered the main clinical outcome.

Secondary endpoints included the development and timing of disease recurrence, as well as the relationship between comorbidity scores and these clinical outcomes. Additionally, the prognostic value of comorbidity scores for overall survival was assessed as part of the secondary endpoints.

All statistical analyses were performed using IBM SPSS Statistics for Windows, Version 28.0 (IBM Corp., Armonk, NY, USA). For continuous variables, results were expressed as mean ± standard deviation and median (min–max); for categorical variables, frequencies and percentages were reported. Normality of distribution was assessed using the Shapiro–Wilk and Kolmogorov–Smirnov tests. For comparisons between two independent groups, either the Independent Samples *t*-test or the Mann–Whitney U test was used, depending on data distribution. For categorical variables, the chi-square test or Fisher’s exact test (when appropriate) was applied. The predictive performance of comorbidity indices for mortality was evaluated using Receiver Operating Characteristic (ROC) curve analysis, and area under the curve (AUC), sensitivity, specificity, positive predictive value (PPV), and negative predictive value (NPV) were reported. Kaplan–Meier survival analysis was used to generate overall survival (OS) and progression-free survival (PFS) curves, and group differences were tested using the log-rank test. The effects of risk factors on survival were evaluated using univariate and multivariate Cox proportional hazards regression analyses, with results presented as hazard ratios (HR) and 95% confidence intervals (CI). A *p*-value of <0.05 was considered statistically significant for all tests.

## 3. Results

A total of 130 female patients aged 50 years and above with a diagnosis of ovarian cancer were included in the study. Median age of 60.5 years (range: 50.0–83.0). The vast majority of patients were postmenopausal (92.3%), and 73.1% had at least one comorbidity.

In terms of performance status, 51.5% of patients had an ECOG score of 0, 34.6% had a score of 1, and 13.9% had a score of ≥2. The mean comorbidity scores were as follows: CCI, 4.8 ± 2.0; CIRS-G, 6.3 ± 2.1; ACE-27, 2.2 ± 0.8; Elixhauser Comorbidity Score, 4.2 ± 4.3; Elixhauser Percentile Score, 78.5 ± 4.0; ICED, 0.8 ± 0.6; and FCI, 1.7 ± 0.8.

Disease recurrence was observed in 39.2% of patients. The median follow-up was 19.4 months (range: 0.8–129.0) and the median time to recurrence was 11.4 months (range: 0.7–53.2). The overall mortality rate was calculated as 46.2%. Table 1 summarizes the demographic, clinical, and comorbidity characteristics of the patients included in the study.

Patients were categorized into two groups based on survival status. Those who died during follow-up were classified as exitus-positive [EX (+)], while those who were alive or confirmed to be alive at the time of last contact were classified as exitus-negative [EX (−)]. All subsequent analyses were conducted based on this classification.

Patients in the EX (+) group had a significantly higher age at diagnosis compared to the EX (−) group (64.2 ± 9.0 vs. 60.4 ± 7.4 years; *p* = 0.033). All patients in the EX (+) group were postmenopausal, whereas 11.2% of patients in the EX (−) group were premenopausal (*p* = 0.025). Additionally, ECOG performance scores were significantly higher in the EX (+) group (*p* = 0.001).

Regarding comorbidity scores, patients in the EX (+) group had significantly higher values for the CCI (5.7 ± 2.4 vs. 4.4 ± 1.6; *p* = 0.001), CIRS-G (7.0 ± 2.3 vs. 6.1 ± 2.0; *p* = 0.041), ACE-27 (2.3 ± 0.5 vs. 2.1 ± 0.9; *p* = 0.003), ICED (1.1 ± 0.7 vs. 0.7 ± 0.6; *p* = 0.017), and FCI (1.9 ± 1.0 vs. 1.5 ± 0.7; *p* = 0.038). No statistically significant differences were observed between groups for the Elixhauser comorbidity score or its percentile version (*p* > 0.05) (Table 2, Supplementary Figure S1).

**Figure 1:**
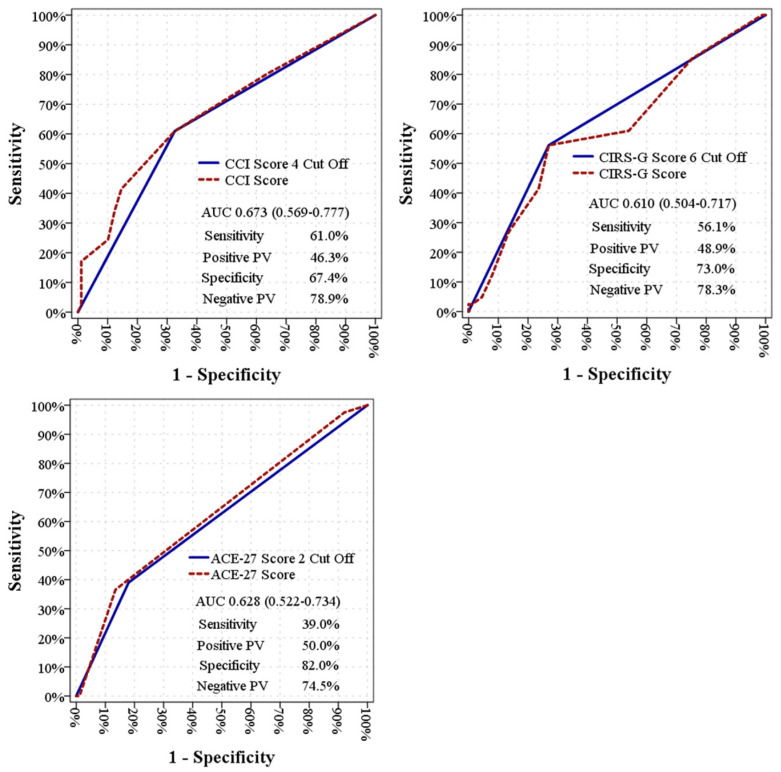
ROC curves of the Charlson Comorbidity Index (CCI), Cumulative Illness Rating Scale-Geriatric (CIRS-G) and Adult Comorbidity Evaluation-27 (ACE-27) scores in predicting overall survival. AUC, sensitivity, specificity, positive predictive value and negative predictive value are presented for each comorbidity score according to defined cut-off values.

According to ROC curve analysis, CCI demonstrated the highest discriminative power in predicting mortality (AUC: 0.673; 95% CI: 0.569–0.777; *p* = 0.002). Using a cutoff value of >4 for CCI, sensitivity was 61.0%, specificity was 67.4%, positive predictive value was 46.3%, and negative predictive value was 78.9% (Table 3). The AUC for CIRS-G was 0.646 (*p* = 0.008), and for ACE-27, 0.628 (*p* = 0.019). The discriminatory power of ACE-27 at the cutoff value of 2 did not reach statistical significance (*p* = 0.054) (Figure 1, Supplementary Tables S1 and S2).

The incidence of recurrence was significantly higher in EX (+) patients compared to EX (−) patients (54.0% vs. 32.6%; *p* = 0.022). Moreover, the mean time to recurrence was significantly shorter in the EX (+) group (12.8 ± 13.1 vs. 16.9 ± 12.1 months; *p* = 0.011). No significant difference was observed between the two groups in terms of follow-up duration (*p* = 0.904)

In univariate Cox regression analysis, higher scores on CCI (HR: 1.276; *p* = 0.000), CIRS-G (HR: 1.149; *p* = 0.028), ACE-27 (HR: 1.388; *p* = 0.023), Elixhauser (HR: 1.081; *p* = 0.035), ICED (HR: 1.804; *p* = 0.012), and FCI (HR: 1.352; *p* = 0.039) were all significantly associated with shorter survival (Table 4). However, in the multivariate model, only the CCI remained an independent prognostic factor for survival (HR: 1.271; 95% CI: 1.118–1.445; *p* = 0.000) (Table 5).

**Figure 2:**
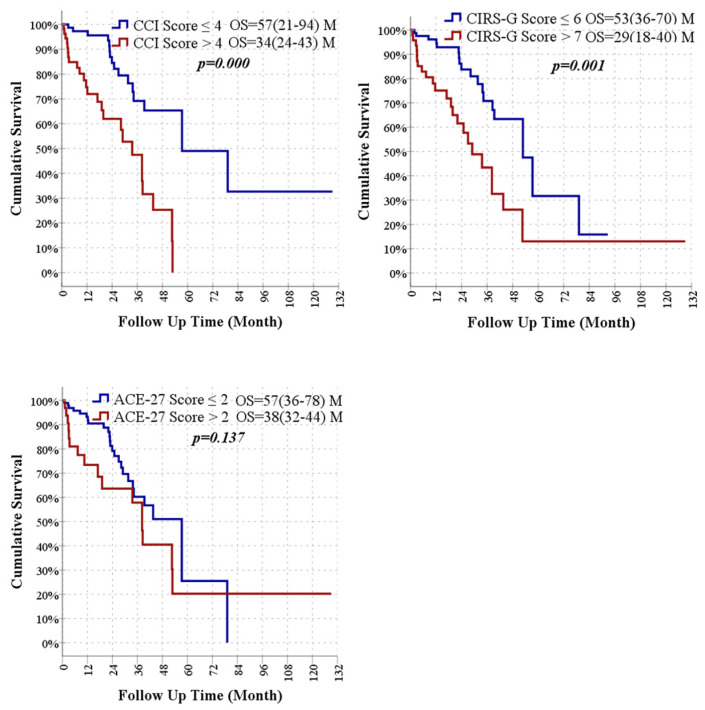
Kaplan–Meier survival curves for overall survival based on CCI, CIRS-G, and ACE-27 score thresholds.

According to Kaplan–Meier analysis, patients with a CCI score > 4 had significantly shorter survival compared to those with CCI ≤ 4 (33.6 vs. 57.3 months; *p* < 0.05). Similarly, patients with a CIRS-G score > 7 had shorter survival than those with CIRS-G ≤ 6 (29.0 vs. 52.8 months; *p* < 0.05). Although survival time was also shorter in patients with ACE-27 > 2 compared to those with ACE-27 ≤ 2 (38.3 vs. 57.3 months), the difference was not statistically significant (*p* > 0.05) (Figure 2).

## 4. Discussion

This study is one of the few that comparatively evaluates the relationship between seven widely used comorbidity indices and survival/mortality in ovarian cancer patients aged 50 years and above. The findings are particularly notable in highlighting the CCI as a strong prognostic indicator, given its high discriminative power in ROC analysis and its retention as an independent predictor of survival in multivariate modeling. While CIRS-G and ACE-27 were significantly associated with overall survival in univariate Cox analysis, they lost their statistical significance in multivariate models. The observation of more frequent and earlier recurrence in patients who experienced mortality suggests that comorbidity burden may directly influence clinical disease trajectory. These findings support the potential utility of comorbidity-based risk stratification as a systematic and predictive tool in guiding treatment decisions for elderly patients with ovarian cancer.

It has been widely acknowledged that elderly ovarian cancer patients are underrepresented in clinical trials, and as a result, treatment decisions are often based on data derived from younger populations. The significant impact of comorbidity scores on survival, as demonstrated in this study, clearly highlights the need for patient-specific data generation and the development of age-adapted treatment guidelines for older adults [6].

In a study by Liontos et al., overall survival in ovarian cancer patients aged ≥70 was found to be shorter than in younger patients; however, this difference was attributed not to age itself but to differences in treatment and surgical strategies. Elderly patients were more frequently treated with neoadjuvant chemotherapy and single-agent carboplatin, while primary cytoreductive surgery was less commonly performed. These findings underscore the importance of basing treatment decisions on individual patient factors rather than chronological age [3]. Consistent with this, the literature indicates that older patients with advanced-stage disease may be excluded from standard oncologic treatments due to increased comorbidity burden, polypharmacy, or perceived frailty [7]. These findings collectively suggest that treatment planning should prioritize functional status and treatment suitability over age alone.

The Geriatric Vulnerability Score (GVS), developed by the GINECO group for patients over 70 years of age with advanced-stage ovarian cancer, has highlighted the prognostic significance of geriatric variables in this population. In this prospective study, patients who met three or more frailty criteria—including low serum albumin levels, impaired Activities of Daily Living (ADL), impaired Instrumental Activities of Daily Living (IADL), lymphopenia, and elevated anxiety/depression scores as measured by the Hospital Anxiety and Depression Scale (HADS)—had significantly shorter survival and lower treatment completion rates [8]. Other studies have also underscored the impact of patient-specific factors in the management of ovarian cancer. Teixeira et al. found an inverse association between the presence of comorbidities and familial cancer risk, suggesting that comorbidity in older patients may represent an independent prognostic factor irrespective of genetic predisposition [9].

The CCI is a widely used prognostic tool originally developed to predict long-term mortality. By assigning weighted scores to a variety of medical conditions, it has been used across numerous disease populations—including malignancies—to estimate survival. The reliability, sensitivity, and predictive validity of the CCI have been demonstrated in various clinical settings such as surgical, intensive care, trauma, and oncology populations. In oncologic studies, an incremental increase in the CCI score has consistently been associated with reduced survival, reinforcing its value in personalized treatment and follow-up planning in malignant diseases [10].

Several studies focusing specifically on ovarian cancer have confirmed the significant impact of the CCI on both survival outcomes and treatment feasibility. In a large, population-based Danish study, Grann et al. reported that the prevalence of comorbidities among ovarian cancer patients increased from 25% to 35% between 2000 and 2011. They demonstrated that the CCI had a statistically significant impact on five-year survival, independent of age and year of diagnosis [11]. Similarly, in a propensity score–matched analysis, Zhao et al. showed that higher CCI scores in elderly ovarian cancer patients were associated with reduced likelihood of undergoing surgery and completing systemic therapy; however, when systemic treatment was completed, survival outcomes were comparable to those of younger patients [12].

In an analysis by Park et al. involving patients aged ≥65 years who underwent cytoreductive surgery for advanced-stage ovarian cancer, postoperative complications were primarily associated with frailty indicators such as higher CCI scores, American Society of Anesthesiologists (ASA) physical status classification, polypharmacy, and low serum albumin levels [13]. In a multicenter study by Dion et al., despite having similar tumor characteristics, women aged ≥75 were found to receive combined surgery and chemotherapy less frequently than those aged 70–74, with lower rates of bowel resection and significantly less frequent use of bevacizumab. Treatment strategies were observed to vary not only by age but also according to frailty level. For example, patients with a modified Charlson Comorbidity Index (mCCI) > 3 underwent less-complex surgeries and had relatively lower rates of postoperative complications. However, median overall survival in this subgroup declined to as low as 28 months, and the five-year survival rate was significantly lower (30% vs. 62%; *p* < 0.001). These findings highlight the critical importance of using objective frailty assessments—rather than chronological age alone—to guide treatment planning and improve prognosis in elderly ovarian cancer patients [14]. In another large cohort study from Denmark, comorbidity burden as measured by the CCI was shown to significantly reduce survival, particularly in patients with FIGO stage II–III disease [15]. Similarly, in an analysis by Vranes et al. using the SEER-Medicare dataset, the ovarian cancer–specific comorbidity index (OCCI) was significantly associated with both overall and cancer-specific survival, whereas the CCI was predictive of overall survival only [16]. These findings from the literature support the role of comorbidity assessment as a key element in guiding treatment decisions and predicting survival outcomes in elderly patients with ovarian cancer. Consistent with these results, our study also demonstrated that patients with higher CCI scores had significantly shorter survival, higher recurrence rates, and that CCI remained an independent prognostic factor in multivariate analysis. Collectively, these findings reinforce the value of the CCI as a reliable clinical tool to guide personalized treatment strategies in older patients with ovarian cancer.

In the study by Mallen et al., involving patients aged ≥70 years with advanced-stage epithelial ovarian cancer, higher CIRS-G scores were not found to significantly influence the likelihood of undergoing surgery, completing chemotherapy, or survival. Instead, suboptimal cytoreduction, platinum resistance, and incomplete adjuvant therapy were identified as the primary factors negatively affecting survival in the elderly population. These findings suggest that tumor biology and treatment tolerance may have a greater impact on survival than comorbidity burden in older patients [17]. In contrast, our study identified a statistically significant association between higher CIRS-G scores and both shorter survival and increased mortality. However, CIRS-G did not emerge as an independent prognostic factor in multivariate analysis. This suggests that while CIRS-G may be associated with some clinical outcomes, its prognostic strength may be more limited compared to other indices such as the CCI.

The ACE-27 score has been evaluated in a limited number of studies, primarily for its utility in predicting perioperative complications and survival in elderly patients undergoing cytoreductive surgery for advanced epithelial ovarian cancer. Zhao et al. reported that patients with higher ACE-27 scores had significantly higher rates of Clavien-Dindo grade ≥ III complications and unplanned intensive care unit admissions, and also demonstrated markedly reduced overall survival. In our study, ACE-27 was significantly associated with survival in univariate analysis; however, it did not retain statistical significance in multivariate modeling. These findings suggest that ACE-27 may have limited value as an independent prognostic indicator [12].

In a retrospective cohort study by Jørgensen et al., patients aged ≥70 years with severe comorbidities were significantly less likely to undergo cytoreductive surgery and receive standard platinum-taxane–based chemotherapy. Notably, among patients who were able to receive optimal treatment, advanced age alone did not independently affect survival outcomes. In contrast, an ASA physical status score ≥ 3 was significantly associated with worse overall and progression-free survival [18]. These findings reinforce the notion that treatment decisions in elderly patients should be guided by functional status and comorbidity burden rather than chronological age.

In the study by Filippova et al., despite high rates of frailty among patients aged ≥74 years, cytoreductive surgery was performed safely using a multidisciplinary team approach, with no postoperative mortality and a low rate of major complications (only 8%) [19].

Van Walree et al. reported that only 5% of patients aged ≥75 received standard treatment as outlined in national guidelines, while the majority were managed with modified therapy or supportive care alone. Nevertheless, survival outcomes were found to be comparable between those who received standard versus adapted treatment [20]. These findings indicate that individualized treatment strategies can preserve clinical effectiveness in elderly patients.

Accurate identification of elderly and frail patients is critical for predicting treatment response and improving outcomes. In this context, clinical trials tailored specifically to older populations and treatment models based on comprehensive assessment are urgently needed. Moreover, stronger multidisciplinary collaboration between geriatricians, medical oncologists, and gynecologic oncologic surgeons will enhance both pre-treatment risk assessment and post-treatment follow-up [21].

In conclusion, our study demonstrates that comorbidity burden has a significant impact on survival and recurrence in elderly ovarian cancer patients, with the CCI emerging as the most clinically predictive tool among the evaluated scores. While the retrospective design, single-center setting, and limited sample size represent methodological limitations that may affect generalizability, the comparative analysis of multiple comorbidity indices is a notable strength that contributes to the existing literature. Our findings suggest that in the elderly population, incorporating comorbidity-based assessments—rather than relying solely on chronological age—into treatment decision-making has the potential to improve clinical outcomes. Future prospective, multicenter, and large-scale studies are warranted to validate these results and support the development of individualized treatment algorithms for elderly and frail ovarian cancer patients.

## 5. Conclusions

This study demonstrated the prognostic value of comorbidity scores in predicting survival among ovarian cancer patients aged 50 years and above. The observed increase in mortality rates and shortened time to recurrence in patients with a higher comorbidity burden suggest that these parameters may directly influence disease trajectory. Among the scores evaluated, only the CCI was identified as an independent prognostic factor in multivariate analysis, while the others showed significance only at the univariate level. These findings indicate that in older patients, treatment decisions should not be based solely on chronological age but should also incorporate comorbidity-based risk stratification to improve clinical outcomes.

## Figures and Tables

**Table 1:** Baseline demographic, clinical, and comorbidity characteristics of the study population (*n* = 130).

		Min–Max	Median	Mean ± SD/n−%		
Age at Diagnosis		50.0–83.0	60.5	61.6	±	8.1
Height		145.0–189.0	158.0	158.9	±	7.4
Weight		30.0–105.0	68.0	68.6	±	14.7
BMI		11.9–44.4	26.9	27.2	±	5.6
Menopausal Status	Premenopausal			10		7.7%
	Postmenopausal			120		92.3%
Comorbidity	(−)			35		26.9%
	(+)			95		73.1%
Smoking	(−)			113		86.9%
	(+)			17		13.1%
Alcohol	(−)			127		97.7%
	(+)			3		2.3%
ECOG PS	0			67		51.5%
	I			45		34.6%
	II			12		9.2%
	III			5		3.8%
	IV			1		0.8%
CCI Score		3.0–11.0	4.0	4.8	±	2.0
CCI Score	≤4			76		58.5%
	>4			54		41.5%
CIRS-G Score		3.0–14.0	6.0	6.3	±	2.1
CIRS-G Score	≤6			83		63.8%
	>7			47		36.2%
ACE-27 Score		1.0–9.0	2.0	2.2	±	0.8
ACE-27 Score	≤2			98		75.4%
	>2			32		24.6%
Elixhauser Comorbidity Index Score		−5.0–18.0	4.0	4.2	±	4.3
Elixhauser Comorbidity Index %		65.7–87.0	78.7	78.5	±	4.0
Index of Coexistent Disease (ICED)		0.0–3.0	1.0	0.8	±	0.6
Functional Comorbidity Index (FCI)		1.0–5.0	1.0	1.7	±	0.8
Recurrence	(−)			79		60.8%
	(+)			51		39.2%
Recurrence Duration (Months)		0.7–53.2	11.4	15.6	±	12.5
Follow-up Duration (Months)		0.8–129.0	19.4	23.2	±	19.1

**Table 2:** Comparison of clinical and comorbidity variables between patients who experienced exitus (EX) and those who did not.

		EX (−)				EX (+)				*p*	
		Mean ± SD/n−%			Median	Mean ± SD/n−%			Median		
Age at Diagnosis		60.4	±	7.4	59.0	64.2	±	9.0	62.0	**0.033**	^m^
Height		159.0	±	7.2	158.5	158.9	±	8.1	158.0	0.996	^t^
Weight		68.1	±	14.7	68.0	69.7	±	14.9	69.0	0.592	^t^
BMI		26.9	±	5.3	26.8	27.9	±	6.4	27.6	0.386	^t^
Menopausal Status	Premenopausal	10		11.2%		0		0.0%		**0.025**	^X^2^^
	Postmenopausal	79		88.8%		41		100%			
Comorbidity	(−)	26		29.2%		9		22.0%		0.386	^X^2^^
	(+)	63		70.8%		32		78.0%			
Smoking	(−)	75		84.3%		38		92.7%		0.186	^X^2^^
	(+)	14		15.7%		3		7.3%			
Alcohol	(−)	86		96.6%		41		100%		0.551	^X^2^^
	(+)	3		3.4%		0		0.0%			
ECOG PS	0	55		61.8%		12		29.3%		**0.001**	^X^2^^
	I	27		30.3%		18		43.9%			
	II	5		5.6%		7		17.1%			
	III	2		2.2%		3		7.3%			
	IV	0		0.0%		1		2.4%			
CCI Score		4.4	±	1.6	4.0	5.7	±	2.4	5.0	**0.001**	^m^
CIRS-G Score		6.1	±	2.0	6.0	7.0	±	2.3	7.0	**0.041**	^m^
ACE-27 Score		2.1	±	0.9	2.0	2.3	±	0.5	2.0	**0.003**	^m^
Elixhauser Comorbidity Index Score		3.8	±	3.5	4.0	5.0	±	5.7	4.0	0.500	^m^
Elixhauser Comorbidity Index %		78.9	±	3.2	78.7	77.8	±	5.3	78.7	0.476	^m^
Index of Coexistent Disease (ICED)		0.7	±	0.6	1.0	1.1	±	0.7	1.0	**0.017**	^m^
Functional Comorbidity Index (FCI)		1.5	±	0.7	1.0	1.9	±	1.0	2.0	**0.038**	^m^
Recurrence	(−)	60		67.4%		19		46.3%		**0.022**	^X^2^^
	(+)	29		32.6%		22		54%			
Recurrence Duration (Months)		16.9	±	12.1	13.0	12.8	±	13.1	9.8	**0.011**	^m^
Follow-up Duration (Months)		23.2	±	19.7	18.1	23.0	±	18.1	22.6	0.904	^m^

^t^ Independent sample *t* test/^m^ Mann–Whitney U test/^X2^ Chi-square test (Fisher test).

**Table 3:** Diagnostic performance of the Charlson Comorbidity Index (CCI) in predicting overall survival, including area under the curve (AUC) values, sensitivity, specificity, and predictive values for the >4 threshold.

		Area Under the Curve		95% Confidence Interval	*p*
CCI Score		0.673		0.569–0.777	**0.002**
CCI Score 4 Cut Off		0.642		0.538–0.745	**0.009**
		EX (−)	EX (+)		%
CCI Score	≤4	60	16	Sensitivity	61.0%
	>4	29	25	Positive Predictive Value	46.3%
				Specificity	67.4%
				Negative Predictive Value	78.9%

**Table 4:** Univariate Cox regression analysis of comorbidity scores in relation to overall survival.

	Univariate Model		
	HR	% 95 GA	*p*
CCI Score	1.051	0.890–1.240	0.557
CIRS-G Score	1.084	0.944–1.244	0.256
ACE-27 Score	1.130	0.765–1.669	0.539
Elixhauser Comorbidity Index Score	0.970	0.892–1.055	0.472
Elixhauser Comorbidity Index %	1.033	0.944–1.131	0.477
Index of Coexistent Disease (ICED)	1.353	0.878–2.083	0.170
Functional Comorbidity Index (FCI)	1.055	0.769–1.447	0.740
Cox Regression			

**Table 5:** Univariate and multivariate Cox regression analyses of comorbidity scores for overall survival.

	Univariate Model			Multivariate Model		
	HR	% 95 GA	*p*	HR	% 95 GA	*p*
CCI Score	1.276	1.121–1.452	**0.000**	1.271	1.118–1.445	**0.000**
CIRS-G Score	1.149	1.015–1.300	**0.028**			
ACE-27 Score	1.388	1.046–1.842	**0.023**			
Elixhauser Comorbidity Index Score	1.081	1.006–1.162	**0.035**			
Elixhauser Comorbidity Index %	0.920	0.851–0.994	**0.035**			
Index of Coexistent Disease (ICED)	1.804	1.139–2.858	**0.012**			
Functional Comorbidity Index (FCI)	1.352	1.015–1.800	**0.039**			
Cox Regression (Forward LR)						

## Data Availability

The datasets used and/or analyzed during the current study are available from the corresponding author on reasonable request.

## Supplementary Materials

The following supporting information can be downloaded at: https://www.mdpi.com/article/10.3390/medicina62010189/s1, Figure S1: Estimated mortality probabilities by Charlson Comorbidity Index (CCI) and Cumulative Illness Rating Scale-Geriatric (CIRS-G) scores; Table S1: Diagnostic performance of the CIRS-G in predicting overall survival, including AUC values, sensitivity, specificity, and predictive values for the >6 threshold.; Table S2: Diagnostic performance of the Adult Comorbidity Evaluation-27 (ACE-27) score in predicting overall survival, including AUC values, sensitivity, specificity, and predictive values for the >2 threshold.
