# Supplementary material for: Evaluating Comorbidity Scores in Geriatric Ovarian Cancer: A Retrospective Cohort Analysis
Source: Medicina (Kaunas). 2026 Jan 16;62(1):189. doi: 10.3390/medicina62010189 (PMC12844226; doi:10.3390/medicina62010189)
Supplement: Supplementary file 1 [file medicina-62-00189-s001.zip › Supplement Table S2.pdf]

Supplement Table S2: Diagnostic performance of the ACE-27 score in predicting overall survival, including AUC values, sensitivity, specificity, and predictive values for the >2 threshold.

	Area Under the Curve	95% Confidence Interval	p
ACE-27 Score	0.628	0.522 - 0.734	***0.019***
ACE-27 Score 2 Cut Off	0.605	0.497 - 0.714	0.054

		Kontrol Grubu	Vaka Grubu		%
ACE-27	≤ 2	73	25	Sensitivity	39.0%
	> 2	16	16	Positive Predictive Value	50.0%
				Specificity	82.0%
				Negative Predictive Value	74.5%
