# Supplementary material for: Evaluating Comorbidity Scores in Geriatric Ovarian Cancer: A Retrospective Cohort Analysis
Source: Medicina (Kaunas). 2026 Jan 16;62(1):189. doi: 10.3390/medicina62010189 (PMC12844226; doi:10.3390/medicina62010189)
Supplement: Supplementary file 1 [file medicina-62-00189-s001.zip › Supplementary Table S1.pdf]

Supplementary Table S1: Diagnostic performance of the CIRS-G in predicting overall survival, including AUC values, sensitivity, specificity, and predictive values for the >6 threshold.

		Area Under the Curve		95% Confidence Interval	p
CIRS-G Score		0.610		0.504 - 0.717	***0.044***
CIRS-G Score 6 Cut Off		0.646		0.541 - 0.750	***0.008***
		Kontrol Grubu	Vaka Grubu		%
CIRS-G Score	≤ 6	65	18	Sensitivity	56.1%
	> 6	24	23	Positive Predictive Value	48.9%
				Specificity	73.0%
				Negative Predictive Value	78.3%
