# Supplementary figures and images for: Evaluating Comorbidity Scores in Geriatric Ovarian Cancer: A Retrospective Cohort Analysis
Source: Medicina (Kaunas). 2026 Jan 16;62(1):189. doi: 10.3390/medicina62010189 (PMC12844226; doi:10.3390/medicina62010189)

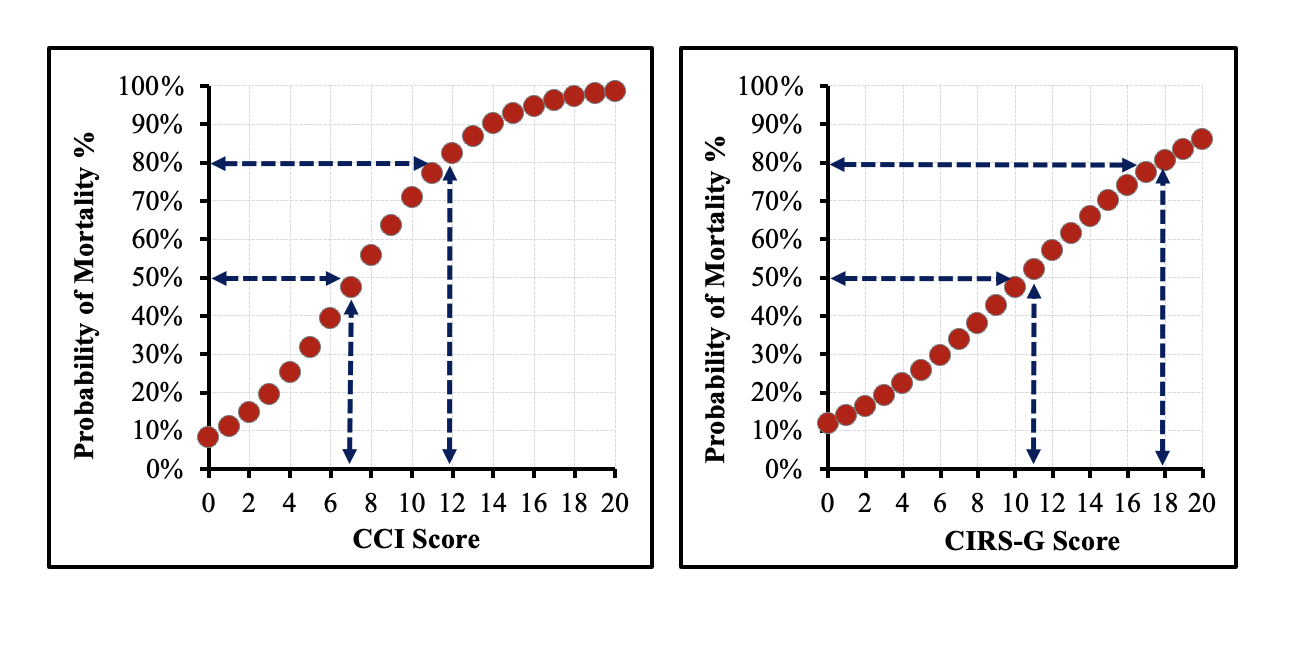

Supplement: Supplementary file 1 [file medicina-62-00189-s001.zip › supp figure S1.png]
